# Operations on the Larynx and Trachea

**Published:** 1909-01-09

**Authors:** 


### Anæsthetics.

### OPERATIONS ON THE LARYNX AND TRACHEA.

To anæsthetisation for the above, the general recommendations given when considering operations on the nose and naso-pharynx (The Hospital, November 21, 1908) are in the main applicable.

Ether is seldom admissible, and even A.C.E. may cause inconvenient or dangerous cough and turgescence. Regard must be given to the amount of dyspnœa already present; if it be very marked, and especially if also dependent on obstruction rather than on reflex irritation it is unsafe to give a general anæsthetic at all. For laryngotomy or tracheotomy local anæsthesia may be employed, especially in adults, and when chloroform is contraindicated; the patient, if nervous or unruly, must be secured from movement by the help of assistants or mechanical means.

If a general anæsthetic is administered for these or for some endo-laryngeal operations, it should not be pushed further than is necessary to avoid struggling and straining. A local anæsthetic may often be used in combination with much advantage, as, for instance, in the removal of papillomata in children; hyperæmia and irritability are diminished, and less general anæsthetic is needed.

For some operations on the larynx, also in the mouth and nasopharnyx preliminary laryngotomy or tracheotomy may be required, especially when there is impediment to breathing, or free hæmorrhage is expected. The larynx should be plugged above the opening, even when a Trendelenburg's tube is used. This is provided with a tampon (inflating, or compressed sponge) intended to prevent blood from running down the trachea, but which it does not always do. It is made to be connected, after breathing through it has been satisfactorily established, with a flexible tube having a funnel-shaped end covered by lint, upon which chloroform is dropped. This, however, rather hampers respiration, and also prevents ready clearance from mucus of the part in the trachea. Quite satisfactory results are obtained by holding a piece of lint, or a catheter or metal tube (connected with a Junker's bottle) opposite the opening of an ordinary tube. Very little chloroform is usually needed to keep up a sufficiently quiet anæsthesia.

